# Kaizen–Kata, a Problem-Solving Approach to Public Service Health Care in Mexico. A Multiple-Case Study

**DOI:** 10.3390/ijerph17093297

**Published:** 2020-05-09

**Authors:** Manuel F. Suárez-Barraza, José A. Miguel-Davila

**Affiliations:** 1 Department of International Business Management, Universidad de las Américas Puebla (UDLAP), San Andrés Cholula 72810, Mexico; 2 GIDE Group, Faculty of Economics and Business, University of León, 24071 León, Spain; jam.davila@unileon.es

**Keywords:** kaizen, kaizen–kata methodology, case study, health care sector

## Abstract

*Purpose:* Mexico’s public hospitals are experiencing major operational problems which seriously affect the care of Mexican citizens. Some hospitals have initiated efforts to apply the Kaizen philosophy to improve this situation. Therefore, the purpose of this article is to analyze the methodological impact of Kaizen–Kata implementation in Mexican public hospitals that have tried to solve operational problems using this improvement approach. *Design/Methodology/Approach*: The service organization implemented Kaizen–Kata methodology in order to improve one operational problem-process in health care. A case-study approach was used in this research in order to understand the effects of the Kaizen–Kata methodology in solving problems in their operational procedures. *Findings:* Six specific drivers were identified when applying the Kaizen–Kata methodology. Furthermore, the impact on the levels of implementation of the Kaizen–Kata methodology in each of the improvement teams studied was also identified. *Research Limitations:* The main limitation of the research is that only three case-studies are presented thus it is not possible to generalize its results. *Practical Implications (Where Possible):* Other public hospitals can use this specific example as a working guide to solve the operational problems of health systems. *Originality/Value:* A methodology of continuous improvement in manufacturing was imported from the industry sector for application in an operational health care process. The Kaizen–Kata methodology contributed significantly to improving issues involving delays, customer complaints, process reworks and extra-cost, among other effects of operational problems.

## 1. Introduction

Following the change of government in 2018, Mexico’s health system began to bring to light several operational and service problems that had been dragging on before, and that have increased in these years of the new government of Mexico [1]. The structure of the health system dates back to the beginning of the 20th century, known as the Social Protection System in Health (SPSS); it was created in 1905, at the time of President Porfirio Díaz, with the inauguration of the General Hospital of Mexico and in 1917 with the first public institution known at the time as the Department of Public Health. Currently, SPSS is made up of two sectors [2]: (i) the public sector, which includes a variety of social security institutions such as the Mexican Social Security Institute (IMSS), the Institute of Security and Social Services for State Workers (ISSSTE), Petróleos Mexicanos (PEMEX), the Ministry of Defense (SEDENA), the Ministry of the Navy (SEMAR) and other institutions and programs that serve the population, but not including social security, the Federal and State Ministries of Health and even hospitals related to the former Seguro Popular. This public system is broad, large and complex, encompassing 80% of the country’s health coverage; (ii) the private sector, which includes private companies and insurance companies that work in private clinics, offices and hospitals.

Other authors who have studied health in Mexico also state that the status of the health system in Mexico in the 21st century is difficult and complex [3]; not only in terms of the citizen’s perception, but also for managers, health employees, doctors, nurses, specialists and all health workers [1]. The bureaucratic structure of the health system in Mexico makes it complex and difficult to operate. The Secretary of Health and the Secretary of Public Administration have made supervisory visits to specialty hospitals (neurology and pediatrics) in order to understand this new operational reality, which is so difficult and complex, but which exists in many of the country’s public hospitals [1,3]. Doctors, nurses and health employees reported several common problems and failures in the health system in Mexico, both in terms of service operation and lack of medicines—even surgical and specific medical equipment [1]. The bureaucratic structure of the health system in Mexico makes it complex and difficult to operate.

Despite the fact that previous governments have indicated that universal health coverage had been achieved in Mexico, looking for a comprehensive reform to improve the performance of the health system [4]. The reality of the health system, according to Knaul and French [5], is that beyond universal health protection coverage in terms of affiliation and access, there is a very large gap with regard to the efficiency of services and the quality of the health service provided. Other Mexican authors, such as Hernández-Torres and Alcántara-Balderas [6], also criticize the gaps between health coverage and the quality of the health service provided. These authors’ criticisms endorse the importance of service quality beyond full coverage.

In this context, there are three major challenges facing SPSS Mexico. Two of them are at the macro level of the entire health system: (i) strengthening the integration of the health care structure and, of course, ensuring a single “package” of health services for all citizens [5]—a complex issue in such a fragmented health system; (ii) stabilizing the processes of purchasing and supplying drugs in the country’s public hospitals. The third major challenge is at the micro level, as a result of the operational problems that have gone unaddressed over the years. Operational problems emerge on a daily basis, impairing the operation of public hospital health services.

This research focuses on the micro challenge of operational problems in the health system. The literature review [7] has shown over the years that techniques and tools related to Lean Thinking, Six Sigma or Kaizen have been applied in the health sector in other countries [8,9]. Thus, research has shown that these techniques have positive impacts on health processes and services: for Lean Thinking, work such as that of Kohlberg et al. [10] or Pedler and Abbot [11]; for Six Sigma Talgo-Taner et al. [12] and for Kaizen, application works in Mexico such as that of Suárez-Barraza et al. [13] or González-Aleu et al. [14]. In fact, Masaaki Imai [15] commented in a forum aimed at health professionals: “Today Health services, Clinics, Hospitals and enterprises around the world in all industries use Kaizen principles and methods to improve patient outcomes, quality, productivity, speed and both patient and service provider satisfaction”.

Going into detail, the academic literature pertaining to these management applications demands a deeper understanding of the specific implementations that have been carried out in hospitals to solve operational problems through Kaizen. Based on this “gap” in the literature of the few studies that have been carried out on the application of methodologies to solve operational problems in the health sector, the purpose of the research was established: to analyze the methodological impact of the implementation of Kaizen–Kata in Mexican public hospitals that have tried to solve these operational problems under this improvement approach. The research questions governing this study are:

- RQ1: How is Kaizen–Kata methodology applied in the resolution of operational problems in public hospitals in Mexico?
- RQ2: What is the level of application of the different steps of Kaizen–Kata methodology in its implementation when solving operational problems?

The answers to these research questions can be found in the results obtained after the implementation of Kaizen–Kata methodology in three public hospitals. Thus, this paper is structured in four main parts. The first part sets out a conceptual framework based on two components: (i) the first one explains the term “Kata” and background methodologies based on the Kaizen philosophy, in addition to studies on the application of Kaizen in the health sector, (ii) The second part explains the methodological section that describes the data collection of the case study. The third part presents the results of the application of Kaizen–Kata in the service organization. The fourth part ends the research article with concluding remarks.

## 2. Literature Review

### 2.1. Kaizen–Kata Definition

Kaizen is the name of an ancient working philosophy coined by Maasaki Imai [16] in 1986 in his well-known book “Kaizen: the key to Japanese competitive success” [16,17]). Over the years, different authors have taken interest in its theoretical construction [18,19], while other authors have advanced towards new approaches for its application and implementation. Such is the case of Mike Rother [20], who wrote the bestselling book “Toyota Kata”, in which the author shows elements or routines behind the resolution of operational problems through setting improvement goals, applying rapid improvement actions and learning from them. Authors such as Lilja et al. [21] clearly consider it an innovation process using the formula of “Define-Test-Feedback-Revise” iterations or loops. Other authors such as Suárez-Barraza [18] have visualized it as a group of macro and micro metaphors from Toyota Motor Corporation, in which standardization, routines and organizational non-routines (the Kata) allow the organization to generate improvements and innovate to solve its operational problems. Suárez-Barraza [22] developed eight steps to a problem-solving routine (the Improvement Kata), capable of tackling the failures that arise in any type of organization. These eight steps are: (1) profiling and/or identifying the problem; (2) determining the effects of the problem; (3) evaluating the current situation of the problem; (4) identifying the root causes of the problem; (5) proposing an Improvement Action Plan; (6) reviewing the results of the application; (7) correcting or, where appropriate, standardizing the improvement actions proposed; and finally, (8) drawing final conclusions.

Finally, Ferenhoff et al. [23] indicate in their findings that Toyota Kata improves employees’ problem-solving skills by linking their efforts to continuously working-process improvement projects; this increases their technical knowledge and management skills.

### 2.2. Kaizen in Health Care

Already in the work of Womack and Jones [24] reference was made to the application of lean thinking and Kaizen philosophy in the health care system. In fact, achieving reasonable response time and acceptable service quality, as well as putting the patient at the center of operations, remains a challenge for today’s health administrations. Authors such as Young et al. [25] and Spear [26] argue that making Kaizen efforts in health systems can help avoid errors, delays, inadequate processes, duplication and all kinds of MUDA (Japanese word that is translated as WASTE. Defined as: any activity that consumes resources and does not add value to the process) in health care process activities. Kohlberg et al. [10], with their pioneering article, emphasize that continuous improvement models help to significantly improve the performance of processes and services in health care systems (specifically those of Sweden in this case). Later, in another seminal article, Dahlgaard et al. [27] provide a definition in Lean in Health Care: “Creating a culture of continuous improvement and employee involvement to reduce unnecessary activities and satisfy patients and stakeholders”.

Two decades have passed, and the literature on the application of Kaizen in health systems has focused on explaining the efforts of Lean Thinking. Some authors such as Bortolotti et al. [28] have found 14 specific factors that increase employees’ problem-solving capacity when using Kaizen in Health Care. The clarity of goals, the degree of difficulty of objectives, the autonomy of work teams and the support of management are critical to the success in the application of Kaizen. On the other hand, Harald Aij and Teunissen [29] evaluate the leadership model of Dombrowski and Mielke [30], where techniques such as Hoshin Kanri, Gemba Kaizen and self-development are techniques that confirm sustainable applications of Kaizen in Health Care. Another group of Brazilian authors, such as Coehlo et al. [31], presents a case study in which the performance improvement in space requirements was 75% and the reduction in waiting time for care was from 2 h to 30 min. Coelho et al. [31] also point out that Lean Kaizen efforts can eliminate at least three hours of overtime per day.

More recently, Abdallah and Alkhaldi [32] identified four Lean bundles related to the application of these techniques in the health care sector. These four bundles are: total quality management and Kaizen; human resource management training and employee empowerment; just-in-time (JIT), focused on inventories and supply chain; and finally, total productive maintenance (TPM), focused on the maintenance of biomedical equipment.

## 3. Research Methodology

In order to strengthen the robustness of the research, as well as to answer the two research questions posed, it was decided to follow a qualitative methodology focused on multiple case studies [33]. This type of research has the following characteristics: (i) an individual point of view is obtained which provides reality close to the object and where the researcher is the instrument; (ii) it studies a limited number of people or particular cases; and (iii) it is based on rich descriptions that facilitate a profound and detailed analysis, rather than generalizations [34]. According to Yin [33], studying a given phenomenon from a qualitative and case study perspective allows us to explore possible causal reasons for the phenomenon in depth by understanding how and why it occurs; for this reason, this methodological approach was used in this research. Therefore, in order to study the application of Kaizen–Kata in the service processes of public hospitals, the role of non-intrusive participative observer was assumed with each hospital selected.

For this, the “theoretical sampling” criterion [4,35] was chosen, which—unlike the statistical sample concept—refers to a type of purposeful sampling in which the researcher selects an individual based on his broad potential for contributing to the development and testing of theoretical constructs. This process continues with other cases until data saturation occurs or a point is reached at which no more results will be found. The data collection process was carried out between May and December 2019 in three public hospitals in Mexico (see Table 1). In this way, three public hospitals in Mexico were selected by theoretical sampling. The reason for their selection was based on three main arguments: (i) the three hospitals had had at least three years’ experience in quality certifications and application of total quality-management techniques; (ii) the public hospital directors were very open to making changes in their daily operations; (iii) the three hospitals are linked to the strategic projects of the health goals of the federal government of Mexico. Thus, the three hospitals stand out in effort and performance with respect to other hospitals in the area in terms of service quality and response times.

The data collection process for the case studies adhered to the following methods:

Direct Observation. Observing the health environment directly is a priority that even public officials in the current government respect; understanding service processes in the workplace (*gemba*) is key to understanding failures and MUDA of public hospitals [36]. This research was no exception and the workplaces where the processes took place were studied with corresponding attention. As direct observation protocol, emphasis was placed on studying the processes of Accident and Emergency (A&E) management and care of citizens (potential patients) when they go to a general practitioner. In all three case studies, this encompassed offices, health care centers, A&E wards, surgery and storage rooms, among others. In addition, inspections consisted of two weekly visits of 2 h each during the months of May to December 2019. The researchers participated in a non-intervention observer role in order to take notes on the work of each worksite where the operational problem of each team occurred.

Non-intrusive participative observation. A role of “non-intervention” was exercised in the daily management of hospitals [34]. In cases A and C the researchers had the opportunity to participate in some work meetings of the Kaizen–Kata teams at the time of applying the methodology (only as observers). This was helpful to the researchers, as Campbell and Gregor [37] point out, in order to understand the set of problems to be investigated if they themselves become familiar with the object of study and the experiences that lie behind them. Using this method of data collection, 28 field events were carried out during these months, which included work meetings of the Kaizen–Kata teams, presentation of some improvement projects, as well as specific meetings of the leaders of the continuous-improvement teams with some of the employees who applied the tools of the methodology. The role of the researcher during these observations was one of total exclusion from the events observed. On no occasion did he participate by giving a point of view or making a comment. Furthermore, attention was paid at all times to avoid transmitting any verbal communication in the form of gestures of approval or disapproval when any of those observed presented an idea or an argument during meetings.

Documentary analysis. This consisted of reviewing the selected material in the form of documents that each hospital provided for study, having access to work manuals, the government’s website with respect to the Department of Health, administrative manuals, operation manuals and, in the case of hospital A, its ISO 9000-quality manual, as well as documented improvement projects. During the collection of this material, special attention was given to obtaining evidence from different documentary sources referring to a similar set of facts [34]. This process provided essential support in reducing the retrospective bias that generally appears in in-depth interviews, given that managers and/or directors of hospitals go back at least five years to reconstruct events of some management practices, increasing the probability of mistakes being made or their memories failing [38].

In-depth semi-structured interviews. These were held with the staff of each of the three case studies. A total of 14 semi-structured interviews between 1 and 1½ h in length were conducted (see Table 2). Each of the interviews followed a specific semi-structured script, revolving around questions that allowed the interviewee to tell his or her experience, emotions and stories focused on the application of the Kaizen–Kata methodology in each hospital. Each interview was recorded and transcribed no more than two days after it took place. Moreover, all 14 interviews took place in the hospitals of the individuals studied. In practical terms, each interview attempted to understand “how” applications or implementations of Kaizen–Kata were carried out in their hospital and of course, the impact it had in terms of improving patient treatment, response time and medical solutions for each patient and each service. It should be remembered that the stories of these people are linked with their own personal experience of their day-to-day hospital management work and therefore, in line with the research questions, the aim was to understand their daily reality as far as possible [37]. Table 2 shows a summary of all the participants interviewed for the research.

Finally, around 156 pages of a researcher’s diary were written up, including all the notes made on each occasion when the process was monitored; this diary was of supreme importance since it represented a source of information for guiding and adjusting the research when this was necessary.

Once all the data were collected, they were downloaded into a database in which each of the methods and data collected were located [33]. The idea was to maintain a “constant comparison” of the data [35] and to be able to identify common codes from the data obtained [39].

In order to generate a measurement of the impact obtained in the case studies, a radar chart was designed based on an adaptation of the steps of the Kaizen–Kata methodology used by Suárez Barraza [22]. For this particular research, the Kaizen–Kata methodology is defined as a theoretical framework as follows: “A methodology of the Kaizen philosophy represents a constant effort of improvement in daily work, in which an improvement team seeks to identify, analyze and solve the root causes of a problem in an operative process of the organization with the goal of changing its status quo” [22]. In that sense, it reorganized them into seven steps for practical purposes: (1) identification of the problem; (2) effects or consequences of the problem; (3) data collection through the check list; (4) prioritization of the main effects through the Pareto diagram; (5) determination of the root causes (Ishikawa diagram); (6) elaboration of the Improvement Action Plan and its implementation; and finally, (7) standardization of the process.

Each of these steps was analyzed by the eight Kaizen teams (KTs) of the three hospitals, so that each one could evaluate the impact of each step of the methodology in the resolution of the selected operational problems. The assessment was made by rating the steps from 1 (no application) to 5 (high application/effective). The data collection procedure was carried out by distributing and collecting a small questionnaire with the indicated scale; once the task of filling in the questionnaire for all members of each KT was completed, the first author met with them to analyze and discuss the application of the Kaizen–Kata methodology and its impact on the selected operational problems. In addition, some members of these teams were interviewed (the same participants as in the interview mentioned above) to get more information about the impact of the Kaizen–Kata methodology. Table 3 shows the characteristics of each KT that participated in the study. The main reasons for selecting the number of teams were: (i) the size of the hospital, (ii) staff trained in quality systems and Kaizen, (iii) staff experience and availability.

## 4. Case Studies Application of Kaizen–Kata Methodology

As indicated in the methodology section, the research was carried out in three public hospitals in Mexico. At the time of the study, each hospital was in a different phase of applying the Kaizen–Kata methodology, while aiming to solve operational problems in the service processes of their hospitals. Case A had made the most progress and gained the most experience in the implementation; case B was at a similar stage, but with less time of application. And finally, case C was a smaller hospital which had started a pilot test of the methodology with several processes in its hospital. To explain this section, each step of the methodology applied is described briefly for each hospital, with examples of each of the steps.

As can be seen in the development and implementation phases of Kaizen–Kata methodology, its application took place from November 2018 to January 2020, during different periods for each hospital. The continuous-improvement project was implemented in four phases, supported at all times by a specialist in Kaizen–Kata methodology (first author): (i) phase of preparation and identification of the problems; (ii) phase of measuring the current situation of the problems; (iii) phase of prioritization of the effects and search for root causes; and, (iv) Improvement Action Plan and its implementation.

### 4.1. Preparation for Kaizen–Kata Methodology Application and Identification of the Problem and Its Effects

During the preparation phase, three main actions were carried out: (i) elementary diagnosis of the current situation of each hospital in terms the development of Kaizen–Kata application; (ii) training seminars in each hospital about the Kaizen philosophy; and, finally, (iii) training of KTs who held their first sessions to identify the operational problem to be improved.

The initial diagnosis in each case showed failures and errors in public services: for example, long waiting times for patients to be treated or mismanagement of resources and inventories. Therefore, based on different cases of application in hospitals in other countries, the implementation of Kaizen–Kata methodology was recommended to improve the performance of the three public hospitals studied [10,40].

The second action was the realization of 20 h of training and coaching in an experimental seminar of Kaizen–Kata. The purpose of the seminar was to lay the foundations of the necessary knowledge of the Kaizen philosophy and the steps of application of Kaizen–Kata methodology, as well as to create KTs in a formal way during the seminar. The seminar participants were managers, area leaders and operational staff representing key players in the hospital’s processes. Following the Kaizen–Kata seminar training, each participant was provided with the necessary knowledge of the Japanese philosophy, as well as the skills needed to apply Kaizen–Kata methodology, including quality tools, such as check list, a Pareto diagram and an Ishikawa diagram.

Finally, with the Kaizen teams structure in place in each hospital, each team proceeded to identify its operational problems and the effects or consequences of each problem. For the purposes of an objective, unbiased analysis, only the implementation of Kaizen–Kata methodology in three teams (of a total of 8) that successfully concluded the application of the methodology is described; in other words, this research shows in an exemplary way the results of three of the four teams that concluded the application of the Kaizen–Kata methodology in an “optimal way”. Thus, for case A, the KT called “A&E-a” identified the problem of: “failures at the time of admission of emergency patients”. The KT of case B called “patient care” identified the problem of “delays in patient care when they consult a general practitioner”. And finally, the KT from case C called “cystic fibrosis quick-innovation” identified the problem of: “shortage of specific drugs for cystic fibrosis”. After this phase, each KT specifically developed its list of eight possible effects or consequences of each of the identified problems. Table 4 shows these effects of the problem.

### 4.2. Current Situation of Problem and Frequency Data Collection

Each of the three KTs selected for the study recorded the frequencies of the different effects detected. The measurement period was three months for each case, with each effect measured daily. The frequency of incidence sought at all times to reflect how many times the effect occurred, and a member of each team recorded it on a checklist. Data collection was open in each hospital and at no time was it a hidden investigation or one in which other employees felt harassed at the time of data collection. Both the Kaizen–Kata staff team and the KT leaders explained in detail how data collection was to be done. Instead, each KT was urged to be as involved as possible with each employee with the aim of improving or solving the problem, as a result of which every worker who was not on the KT collaborated actively. Table 4 shows the total results obtained from the three months of measurement.

### 4.3. Prioritizing the Effects of the Problem and Finding Its Root Causes

**Figure 1:**
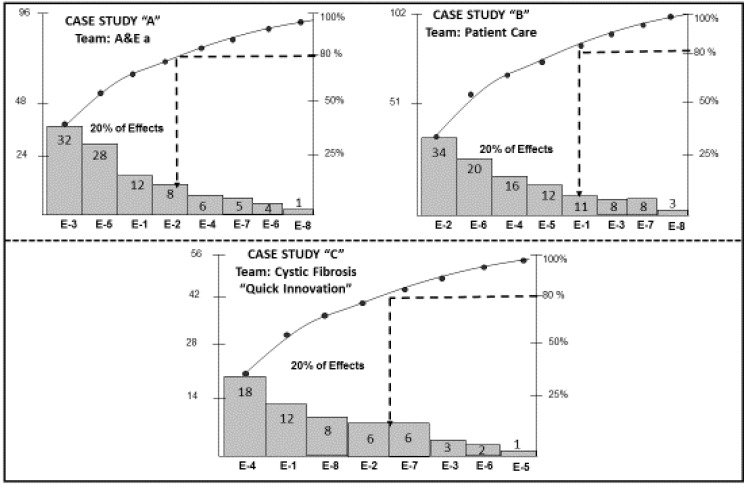
Pareto diagram for each of the KT.

The penultimate step in the methodology applied by each of the three KTs was the construction of Pareto diagrams to prioritize the effects of the problems. Each Pareto diagram allowed each KT to determine the 80%–20% rule of the Pareto principle. The result was the determination of 20% of the priority effects where the true root causes of each problem lies. A total of 8 Pareto diagrams was constructed, one for each team; an example of the three KTs that concluded the application of the Kaizen–Kata methodology with excellence is shown in Figure 1.

As can be seen, 80% of the Pareto principle, i.e., 80% of the errors, are due to four or five effects. For example, for Case A it is four effects, for Case B five effects and for Case C between four and five effects. Within each of the effects are the root causes of the problems studied by each KT.

**Figure 2:**
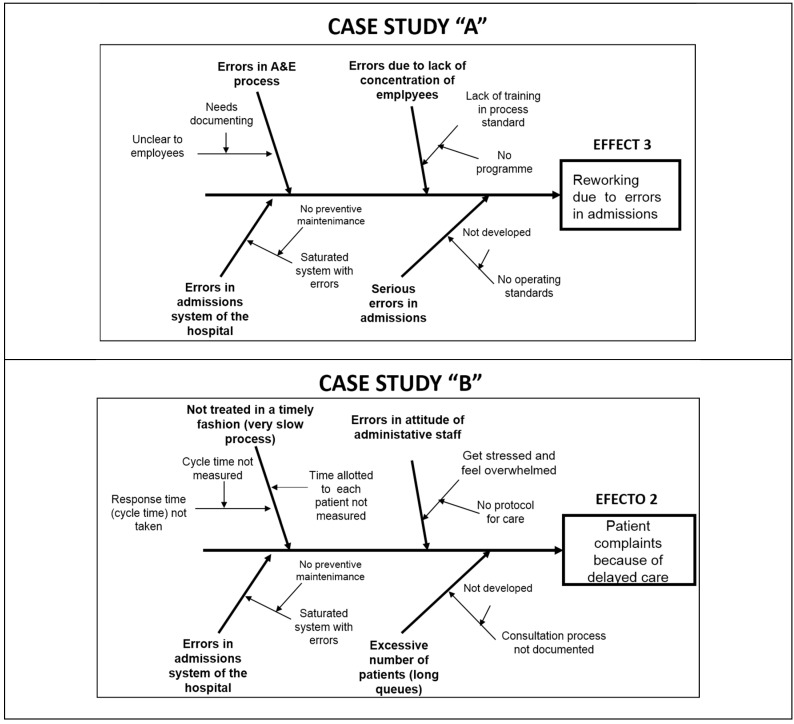

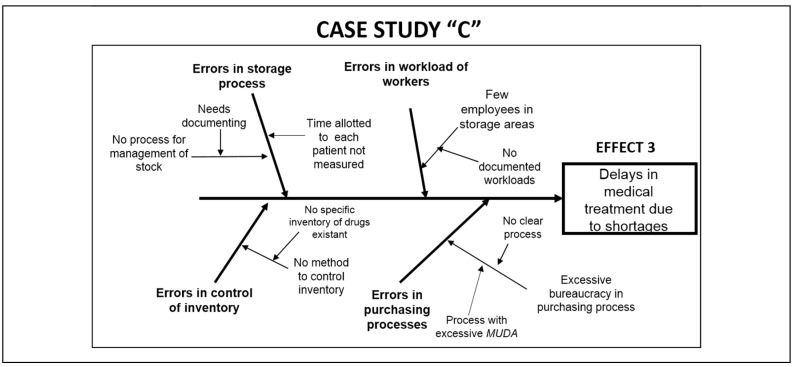
Examples of an Ishikawa diagram for each of the Kaizen Teams.

As a next step, each KT developed the Ishikawa cause–effect diagram in this “relentless” search for root causes. The root causes of the problems are the clues to completely eliminating the problem and its consequences or effects. For this reason, the construction of four or five Ishikawa diagrams allowed the teams to identify the most common and recurrent causes that could be the roots of each problem. Figure 2 shows an example of each of the diagrams constructed for each team demonstrating successful application of Kaizen–Kata methodology.

Globally and from deeper levels of the Ishikawa diagram, at least four types of root causes could be identified: (i) lack of operating standards; (ii) lack of process documentation; (iii) inventory system failures; (iv) failure to train workers in process protocols and customer service so that “quick” service results. Each of these causes was taken into account by the Kaizen–Kata teams to address the last step of the Kaizen–Kata methodology.

### 4.4. Improvement Action Plan and Its Implementation–Standardization

The last step taken by the Kaizen–Kata team was to draw up an improvement action plan with the aim of establishing improvement actions and dates to implement them, as well as specifying persons in charge. The main improvement actions revolved around the documentation of processes, the definition of operating standards once the processes were identified, the training of personnel for new processes and formulating protocols for customer service and attention. For the Kaizen–Kata teams, the construction of a detailed plan which helps them to eliminate operational problems of their hospitals is a key piece of management for improving working conditions; in fact, for the leaders of the Kaizen–Kata teams it was found to be very “strange” to work with this new perspective.

However, the implementation took approximately three to five months depending on the equipment and the hospital. The result was successful in virtually all teams, with 95%–100% implementation (Table 5).

## 5. Findings and Discussion

Based on the results of the implementation of Kaizen–Kata methodology in these three public hospitals, it can be stated that there was a successful application in health service processes in each of the cases studied. Practically all the Kaizen–Kata teams in public hospitals solved their operational problems; 50% lowered the level of incidents of operational effects, while the other 50% completely eliminated operational problems successfully (see Table 3). In accordance with the results from Table 3, the four teams that managed to conclude the methodology (the first three of which with excellent results) were: (1) the A&E team from case A; (2) the cystic fibrosis team, “high-calibre specialists” from case A; (3) the patient care team from case B; and finally, (4) the Cystic Fibrosis team, “Quick Innovation” from case C. Taking into account that Kaizen–Kata methodology was applied by 8 teams in 3 public hospitals, 50% concluded with successful results. On the other hand, the fact that 50% of Kaizen teams that did not conclude the methodology, does not mean that they were not successful; on the contrary, they continue working on the steps that remained of the Kaizen–Kata methodology (see Table 3). In fact, of the four teams that were unfinished, the average compliance with the methodology was 95.25%. In addition, each Kaizen team that had not completed by the time the results were documented reached advanced steps in implementing the Improvement Action Plan, either in reviewing the implementation results or in searching for root causes.

The answer to the first research question posed about how Kaizen–Kata methodology is applied in the resolution of operational problems in public hospitals in Mexico is based on the procedure carried out and the empirical data obtained in the case study of the three hospitals. Of the eight Kaizen–Kata teams studied, at least three of them achieved successful results, while another two finished their improvement projects by reducing the incidences or frequencies of operational problem effects. At the time of documenting the research, four of the eight teams had finished and the other four had remained at the step of searching for root causes, developing an action plan for improvement or verifying the results to standardize the process (steps 5, 6 and 7).

It is verifiable that all Kaizen–Kata teams in public hospitals experienced a form of systematic and continuous improvement which gave them a guiding light in the “sea” of operational problems that these hospitals in Mexico experience. The successful application in solving operational problems in public hospitals of techniques and tools focused on Kaizen philosophy is also corroborated in the literature in different countries such as the USA, Sweden, the UK, Germany and Egypt, among others [28,29,32,41,42]. For this reason, at least six critical drivers were identified during the application of Kaizen–Kata methodology:

1. Effective and committed leadership from the General Directors of the hospitals and the middle management of each public hospital (section heads, area heads, internists).
2. Exhaustive on-the-job or in-house training of the techniques and tools specific to the steps of Kaizen–Kata methodology. This is not an easy aspect to implement, as there is no culture of these techniques, and employees are not engineers. This driver allowed a profound knowledge of each technique and tool studied.
3. Creation of a network of improvement teams called Kaizen–Kata, which allowed a space for dialog on improvement, and a comprehensive and participatory training forum to eliminate problems that arose on a daily basis.
4. Maintaining the specific follow-up of each Kaizen–Kata improvement project through the assistance of a specialized consulting, two-person staff team appointed in each hospital.
5. Disciplined implementation of Kaizen–Kata methodology, applied strictly step-by-step for the resolution of public hospital problems in the “action trench” (in the *gemba* [workplace]), confirming the work of Bortolotti [28] and Ishijima et al. [42]. This involved complete redesign of each public hospital’s strategy, moving from a reactive and “complaining” vision to a much more proactive vision of improvement and change.
6. The application of the Kaizen–Kata methodology allowed the change from a work routine of “simple” execution of operational process activities to a work routine with learning where continuous improvement and problem-solving are part of the day to day procedures. This new way of working was perceived by the employees of public hospitals as “strange”. However, it gradually became their new working paradigm (execution and continuous improvement).

The literature confirms the presence of several of these critical drivers indicated; however, as a vital contribution of our research it was contrasted that in a work culture such as that of the Mexican public health sector, the improvement teams integrated in Kaizen–Kata methodology worked more as mechanisms to promote change and improvement, compared to other workers (protesters and gossipers who were prone to blame hospital directors), without realizing that they themselves were generating the change. In addition, having a simple, clear and easy to apply improvement methodology (i.e., specific steps) clarified the path of change of old management practices.

**Figure 3:**
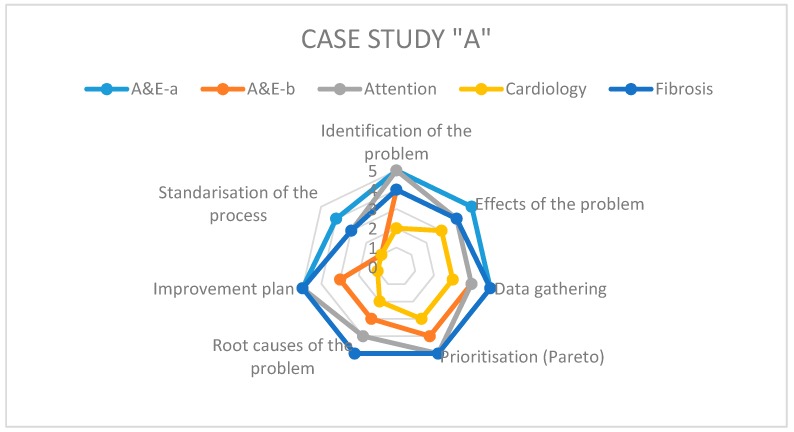
Radar Graph of case study A.

**Figure 4:**
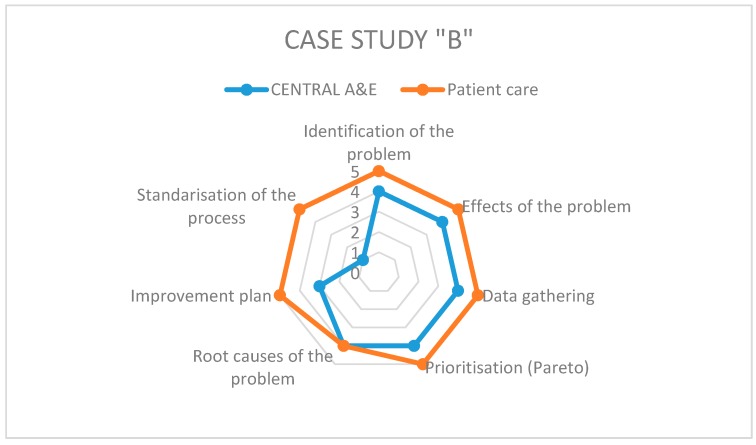
Radar graph of case study B.

**Figure 5:**
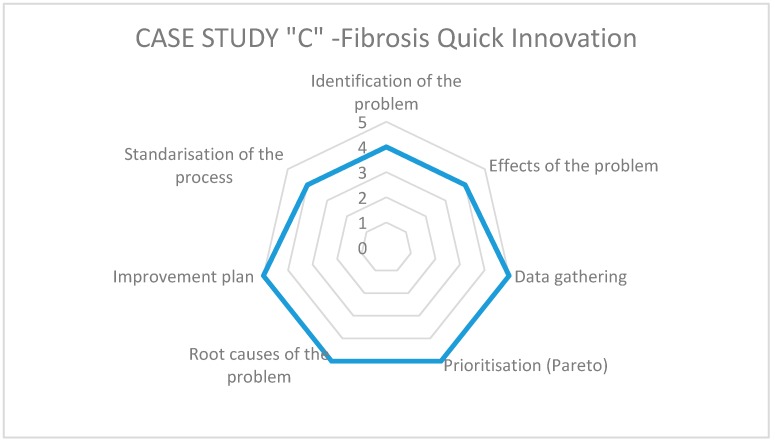
Radar graph of case study C.

On the other hand, to answer the second research question regarding the level of application of the different steps of Kaizen–Kata methodology in its implementation when solving operational problems, we rely on the specific questionnaire pertaining thereto. Each of the teams studied presents differences in the implementation of the methodology according to the working environment of each hospital and the level of understanding of the methodology by each team in each hospital. Figure 3, Figure 4 and Figure 5 show the results of the radar graphs of the 8 Kaizen–Kata teams studied.

As can be seen in the hospital of Case A, of medium-large size, it had the capacity to implement more Kaizen–Kata equipment; in total, there were five teams in different areas of the hospital, able to apply the Kaizen–Kata methodology in varied ways. There were Kaizen–Kata teams such as the “A&E a” and “Cystic Fibrosis” teams which achieved 4 or 5 points (high effective application) in most steps of the methodology. In this type of successful team, the most difficult step to achieve once implemented was the standardization of improvement activities; this is probably due to the resistance to change of bureaucratic structures in public hospitals.

The other three Kaizen–Kata teams shown in the graph had problems from the early stages or when developing the tools in the Ishikawa diagram that look for root causes of the problem. This tool requires a lot of quantitative data (carried over from the previous steps of the methodology) to be able to establish the qualitative cause–effect relationships at the time of construction. A quantitative data collection in the *gemba* allows for a deeper application of the Ishikawa diagram; for this reason, some teams were delayed in the progress of the application of the methodology. These teams had some areas of opportunity at the conclusion of some of the steps of the methodology which delayed the implementation process; for example, the cardiology team skipped the prioritization step (construction of the Pareto diagram) due to an oversight, which caused delays in implementation.

Case B, being smaller in size, only had two Kaizen–Kata teams. The “patient care” team performed well and many of its members were staff, motivated to improve the service provided to patients, for example, by trying to improve waiting times in the pre-consultation room. Therefore, the application of the methodology was successful (mostly five points). The other team (“Central A&E”), with a greater workload due to being in the emergency department, had more areas of opportunity when applying Kaizen–Kata methodology. The step where the process got stuck was the Improvement Action Plan, because several of its actions required some technical investment from the hospital, such as new stretchers, ambulances and medical-support equipment in A&E. The improvement project was left at a point of “work in progress”, with the Kaizen–Kata team trained and motivated.

Case C, the smallest of the public hospitals, implemented a single Kaizen–Kata unit. The team of cystic-fibrosis doctors and nurses worked in a disciplined way to eliminate the problem of “drug shortages” for treating cystic fibrosis, including improving and optimizing their warehouse inventory model—a small, but highly motivated team, as can be seen from their scores of 4–5 on the entire methodology.

Finally, it is important to point out that the literature on the application of Kaizen–Kata is practically non-existent when it comes to describing the operational discipline in application of each step of the methodology by the KTs, that is, how each team applies each step or the “improvement routine”. A poorly applied or “not applied” (skipped) step will result in “failures” or unobserved areas of opportunity in the resolution of operational problems posed [21,43,44]. A noteworthy fact was the observation in some of the steps of the implementation of different inhibitors (elements that block efforts towards improvement) which limited the efficiency of the methodology, such as “resistance to change in other areas of the hospital”, “excessive bureaucracy and regulations”, “disbelief in other areas”, “laziness on the part of some workers” and even “organizational myopia” (indicating that there are no problems in the hospitals, that “everything is fine”).

## 6. Conclusions

This research examines the application of the Kaizen–Kata methodology to solve operational problems in public hospitals in Mexico. Our findings detected six critical drivers in the application: (1) leadership of senior management; (2) operational discipline in the application of the methodology; (3) network of Kaizen–Kata teams; (4) the team of support staff; (5) on-the-job training in the *gemba*; and (6) a shift towards proactive vision and continuous improvement routines for all the employees. We also observed that, of the eight teams studied, at least three produced a “successful application” of four or five points, while others ran up against barriers or inhibitors that hindered the implementation of Kaizen. Thus, according to the results found using the qualitative methodology of the case study, there was no attempt to conclude that Kaizen–Kata methodology is the “total solution” to “all” operational problems of public hospitals. However, the progress in the application of the Kaizen–Kata methodology up to the final levels exhibited by practically all the teams demonstrates that work routines of these public employees, who are used to work related bureaucracy, have changed radically; this is due to having the opportunity of and space for dialog (the Kaizen team) to improve their own operational problems in their daily work.

In fact, as an additional observation, without being able to be empirically contrasted at the time of closing this article, public hospitals that applied Kaizen–Kata methodology seem to be responding better to the coronavirus crisis by having teams prepared with previous learning of a particular methodology. As a proposal to extend the work, a study of this in the future would be interesting. The Kaizen–Kata methodology also explores the possibility of innovating and redesigning processes using other approaches and information technologies such as the implementation of ERP [45]. This topic could also lead to interesting future research in public hospitals.

Finally, a limitation of the work is that results and conclusions of the study cannot be generalized because only three public hospitals were studied; however, this work may represent an implementation guide for other public hospitals in Mexico and other countries that have similar problems in becoming more efficient.

## Figures and Tables

**Table 1:** Selected studies for the application of Kaizen–Kata in public hospitals.

Case	Workplace	Processes Observed	Levels of Continuous Improvement
**Case A**	Public Regional General Hospital of the Federal Social Security in Mexico (Toluca); 1000 beds; 940 employees	A&E (Accident & Emergency) management Medical Care with a Specialist Cardiology specialty process Cystic fibrosis specialty process	Start of the Kaizen–Kata project in February 2019, with integrated project teams Hospital with ISO 9000 certification in the cystic fibrosis process since 2015 Plans for ISO 9000 (ISO-International Organization for Standardization-9000 Quality Management System) certification of the emergency process.
**Case B**	Regional Public Social Security Hospital (San Andrés Cholula); 100 beds; 35 employees	A&E management Medical Care with a Specialist	Kaizen–Kata Project launched in May 2019 to improve emergency and medical care operational issues with integrated project teams.
**Case C**	Medium-sized General Hospital (Tlaxcala); 30 beds; 18 employees	A&E management Medical Care with a Specialist Specialty Flu and Contagiousness Process Cystic fibrosis specialty process	Pilot test (start) of the Kaizen–Kata project from October 2019 for the processes of A&E, medical care, specialty flu and contagion and specialty cystic fibrosis

**Table 2:** Participants interviewed for each selected case study.

Case Selected	Personnel Interviewed
**Case A**	Hospital Director Leader of a Kaizen–Kata Team (A&E team), also leader of the ISO 9000 project. Leader of a Kaizen–Kata Team (Cystic fibrosis team) Specialist Doctor Emergency Technician Administrative employee
**Case B**	7. Hospital Director 8. Leader of a Kaizen–Kata Team 9. Specialist Doctor 10. Emergency Technician 11. Emergency Technician
**Case C**	12. Hospital Director 13. Specialist Doctor 14. Administrative employee

**Table 3:** Details of the Kaizen–Kata teams which participated in the study.

No.	Case Study	Team (1)	Total Team Members	Total Members that Responded (Response Rate%)	Status of Kaizen–Kata Project
1	A	A&E-a	6	100%	Finished with excellent results
2	A	A&E-b	7	98%	Step 5
3	A	Care	8	95%	Step 6
4	A	Cardiology	5	90%	Step 4
5	A	Cystic Fibrosis “High-calibre Specialists”	6	100%	Finished with excellent results
6	B	A&E-Center	7	98%	Step 5
7	B	patient care	7	100%	Finished with excellent results
8	C	Cystic Fibrosis “Quick Innovation”	6	100%	Finished
	**TOTAL**		52	99.5%	

**Table 4:** Identified effects or consequences of each problem and frequencies of each selected problem by Kaizen Team (KT).

TEAM 1	A&E Team-a	
**Problem/Effects**	**Errors in Admissions**	**Frequency**
E1	Complaints from relatives	12
E2	Complaints from the patient	8
E3	Reworking	32
E4	Waste of time	6
E5	Delays in medical processes	28
E6	Employee errors committed while carrying out tasks	4
E7	Waste of material	5
E8	Conflict with management	1
**TOTAL**	**–**	96
**TEAM 2**	**Patient Care Team**	
**Problem/Effects**	**Delays in Care**	
E1	Complaints from relatives	11
E2	Complaints from the patient	34
E3	Reworking	8
E4	Waste of time	16
E5	Delays in medical processes	12
E6	Delays in attending other patients arriving for consultation	20
E7	Complaints from other patients	8
E8	Duplication of tasks by trying to solve “the problem quickly”	3
**TOTAL**	**–**	112
**TEAM 3**	**Fibrosis Team “Quick Innovation”**	
**Problem/Effects**	**Drug Shortages**	**Frequency**
E1	Lack of patient care	12
E2	Complaints from the patient	6
E3	Complaints from relatives	3
E4	Delays in medical treatment	18
E5	Risk of errors in medical procedures	1
E6	Conflict between employees and management	2
E7	Complaints from other patients	6
E8	Duplication of tasks by trying to solve “the problem quickly”	8
**TOTAL**	**–**	56

**Table 5:** Description of the MAP of the Kaizen–Kata equipment studied.

Public Hospital (Case Study)	Kaizen–Kata Team	Participants in Teams	Problem Selected	Improvement Activities (Kaizen)	Kaizen–Kata Process Implementation Rate (%)
Case Study A	A&E A	6	Errors in the admission of patients	Mapping of the emergency process with identification of the MUDA. Elimination of activities that do not add value to the emergency process (unnecessary workload is reduced) Establishment of operating standards for different types of patients arriving at A&E admissions On-the-job training in medical process and service quality Switch to digital logbook (simplified and easy to use)	100% implementation progress
Case Study B	Patient care	7	Delays in patient care	Mapping of the emergency process with identification of the MUDA. Measurement of the real cycle time of the patient care process in the consultation room. Establishment of the ideal time of the “standard” process according to the workload Preventive maintenance to the visiting software	98% implementation progress. Software revision is still pending
Case Study C	Cystic Fibrosis “Quick Innovation”	6	Cystic fibrosis drug shortages	Application of the 5S in the in-house pharmacies Improved process flow by implementing a storage method (first in/first out) New contracts were established with drug suppliers with established delivery times Improved supply and inventory control using Kanban	100% implementation progress

MUDA: Japanese word that is translated as WASTE. Defined as: any activity that consumes resources and does not add value to the process.
